# Versatile subtypes of pericytes and their roles in spinal cord injury repair, bone development and repair

**DOI:** 10.1038/s41413-022-00203-2

**Published:** 2022-03-16

**Authors:** Sipin Zhu, Min Chen, Yibo Ying, Qiuji Wu, Zhiyang Huang, Wenfei Ni, Xiangyang Wang, Huazi Xu, Samuel Bennett, Jian Xiao, Jiake Xu

**Affiliations:** 1 Department of Orthopaedics, The Second Affiliated Hospital and Yuying Children’s Hospital of Wenzhou Medical University, Wenzhou, Zhejiang 325000 China; 2 Molecular Pharmacology Research Centre, School of Pharmaceutical Sciences, Wenzhou Medical University, Wenzhou, Zhejiang 325035 China; 3 Molecular Laboratory, School of Biomedical Sciences, The University of Western Australia, Perth, WA 6009 Australia

**Keywords:** Pathogenesis, Neurophysiology

## Abstract

Vascular regeneration is a challenging topic in tissue repair. As one of the important components of the neurovascular unit (NVU), pericytes play an essential role in the maintenance of the vascular network of the spinal cord. To date, subtypes of pericytes have been identified by various markers, namely the PDGFR-β, Desmin, CD146, and NG2, each of which is involved with spinal cord injury (SCI) repair. In addition, pericytes may act as a stem cell source that is important for bone development and regeneration, whilst specific subtypes of pericyte could facilitate bone fracture and defect repair. One of the major challenges of pericyte biology is to determine the specific markers that would clearly distinguish the different subtypes of pericytes, and to develop efficient approaches to isolate and propagate pericytes. In this review, we discuss the biology and roles of pericytes, their markers for identification, and cell differentiation capacity with a focus on the potential application in the treatment of SCI and bone diseases in orthopedics.

## Introduction

**Fig. 1:**
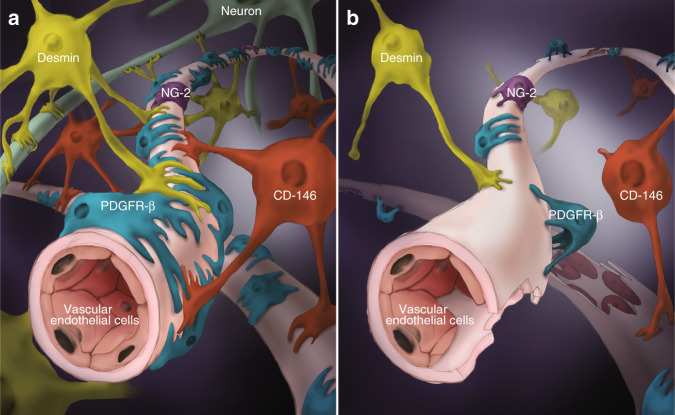
Pericytes in SCI. Schematic representation of the localization, morphology, and coverage of common subtypes of pericytes and vascular endothelial cells in normal spinal cord (**a**) and injured spinal cord (**b**)

The pericyte (also called the Rouget cell or parietal cell) was first discovered by Rouget in 1873, and was named the pericyte by Zimmermann in 1923.^1^ The cytoplasm of the pericyte regularly forms an asteroid protuberance, and pericytes characteristically reside within the vascular basement membrane (which is usually continuous with the endothelial basement membrane), in proximity to, and often encircling, endothelial cells. Pericytes communicate with endothelial cells directly via gap junctions, tight junctions, focal adhesions, and soluble factors.^2,3^ For instance, the connection between the pericyte and the microvascular endothelium of the spinal cord is crucial in maintaining the tight junctions between endothelial cells and the structural integrity of the blood spinal cord barrier.^4^ Endothelial cells enclose the endothelial tubules, and pericytes abut the endothelial cells. Although the basement membrane mostly separates pericytes and endothelial cells, direct intercellular contacts between the two cell types occur at ‘peg-socket’ occlusions and adhesion plaques.^1^ The cell junction and basement membrane-like substance interweave the pericyte, endothelial cells, and the end-feet of the astrocyte (Fig. 1).^5^ Research suggests that pericytes are crucial for blood vessel growth and maturation via the activation of transforming growth factor-β (TGF-β) signaling, and the production of endothelial cell survival factor, angiopoietin 1 (Ang1), as well as by co-occupying endothelial tubules to provide physical stability and support for endothelial tubule function during the initial stage of angiogenesis.^6^ Therefore, the role of pericytes in the formation of the vasculature is of vital significance and is closely related to the form and function of endothelial cells.

Recent research points to the essential role of pericytes in angiogenesis and in the maintenance of vascular supply in SCI and bone fracture healing. Given the broad similarities in potential mechanisms of action of perivascular stem cells (PSC), including direct contribution to cellular regenerate, vasculogenic effects, fibrotic and anti-fibrotic activities, other paracrine effects in the spinal cord and in bone, we survey these two systems to discuss the role and potential application of PSC in the repair of SCI and bone fracture or defect. This review comprehensively defines the subtypes of pericytes, including their potential of stem cells, relationship with vascular homeostasis, and the unique function of the common subtypes of pericytes in the spinal cord and in bone, thereby providing a deeper theoretical basis for the processes of repair following spinal cord injury (SCI) and bone fracture or defect.

## Pericytes have the potential of stem cells

PSC is a term that includes both pericytes and other perivascular progenitor cells with properties of mesenchymal stem cells (MSCs).^7^ The true nature of pericytes, described by characteristics such as their various sources, phenotypes, multipotency, and versatility, remains a controversial area of discussion. The similarities between pericytes and vascular smooth muscle cells are well documented, and whilst together they form the mural cells supporting blood vessels, selective markers, such as CD146, are used to identify phenotypic differences between the two cell types.^8–10^ For example, brain pericytes originate from the mesoderm and neural crest, and can differentiate into neural lineage cells and regulate the neurovascular network.^11^ Pericytes also demonstrate the capacity to differentiate into osteoblasts, chondrocytes, and adipocytes, providing evidence of their mesenchymal lineage differentiation potential.^12,13^ Pericytes and other cell types such as adventitial cells and vascular smooth muscle cells are thought to be responsible for ectopic calcification of blood vessels, heart valves, and skeletal muscle.^12,14^ Pericytes are also considered to be a potential source for cell-based therapeutic applications for the treatment of diseases, and of vital significance is the tissue-specific function and capacity for multilineage differentiation of pericytes.^15^ For instance, recent research is focused on identifying pericyte subsets with the capability to regenerate their tissue microenvironment, and/or maintain a heterologous differentiation potential.^16^ Research has shown that owing to their potential for myogenic differentiation, pericytes may be used to treat Duchenne muscular dystrophy.^17^ In line with this, skeletal pericytes have a greater osteogenic capacity than soft-tissue pericytes.^16^ Moreover, among soft-tissue pericytes, cells expressing CXCR4 were found to be osteoblastic, non-adipocytic precursor cells.^16^ Additional research identified a novel role for pericytes as stem cells capable of modifying the extracellular matrix microenvironment and promoting epidermal tissue regeneration. Pericytes were able to detach from the capillary basement membrane, differentiate into fibroblasts, and form collagen stroma during wound healing.^8,18^ Conversely, the collagen stroma can be fibrotic in chronically inflamed tissue and may form a fibrous stromal tumor, whereby pericytes may be therapeutically targeted.^19,20^ Taken together, the pericyte has stem cell potential, and alteration of the microenvironment will induce pericytes to differentiate towards a particular lineage, to attain various morphological features, and functional properties.^13^ On the other hand, studies of the multipotency of Tbx18-expressing pericytes appear to be inconsistent.^21,22^ Tbx18 was found to be expressed in vascular smooth muscle cells, pericytes, and glomerular mesangial cells, which appear to play a role in the vasculature development of the kidney.^23^ Whilst Tbx18-expressing cells exhibit differentiation properties of MSCs in vitro, they do not appear to function as stem cells in vivo in cell lineage-tracing experiments, such as transgenic mouse models, which indicates that Tbx18-expressing pericytes might not have significant regenerative and fibrogenic properties for therapeutic application.^21,22^ Nevertheless, exploring the source and differentiation cues of pericytes in depth, including the differentiation processes of subtypes of pericytes, are prime areas of research in vascular regenerative medicine as a potential source of the therapeutic cells across many disease entities.

## The subtypes and hallmarks of pericytes

Pericytes share biological functions similar to stem cells and there are several subtypes of pericytes.^1^ Scientists have attempted to find pericyte-specific markers for the classification of the subtypes of pericytes, which has proven challenging. There are several reasons for the difficulty in the classification of pericytes: (1) the isolation of pericytes is difficult; (2) dissociation in the somatic environment may lead to changes in pericyte phenotypes; (3) the differentiation of pericytes makes it difficult to identify the subtypes, and (4) multiple markers can be present on one pericyte. For example, Desmin^−^ pericytes lose CD13-expression during migration away from the blood vessels, so it is speculated that CD13 pericytes and Desmin^+^ pericytes may switch to each other when the microenvironment changes.^24^

Although it is difficult to classify the pericytes, one might attempt to distinguish the different pericyte markers, and their expression levels, by methods, such as immunofluorescence labeling. In this way, one can describe the pericyte subtypes according to their characteristic markers to evaluate their various properties of the function, location, source, and differentiation.

According to the existing research, pericytes express α-smooth muscle actin (SMA), smooth muscle myosin, tropomyosin, nestin, vimentin, desmin, neuron glial antigen 2 (NG2), connexin 43 (Cx43), CD146, endosialin, γ-glutamyl transpeptidase, alkaline phosphatase, protein kinase G (PKG), aminopeptidase A, regulator of G protein signaling 5 (RGS5), platelet-derived growth factor receptor-β (PDGFRβ), ATP-sensitive potassium Kir6.1, CD13, CD248, SUR2, Tbx18, and delta-like 1 homolog (DLK1); but do not express the endothelial cell marker, von Willebrand factor, nor the astrocyte marker, glial fibrillary acidic protein (GFAP).^1,25,26^

As pericytes and other cells could express similar biomarkers, how to distinguish between pericytes and other cells using immunofluorescence staining remains a challenging issue in pericyte research. Most of the existing studies use a variety of pericyte markers to co-stain the pericytes to determine their source, and to distinguish the pericyte subtypes. For example, pericytes could be identified and studied in relation to tissue-specific injury and angiogenesis using differential nestin/NG2 staining. Meanwhile, PDGFR-β, NG2, and CD146 have also been applied to immunofluorescence staining to distinguish pericytes from other cells.^27,28^ Therefore, it is feasible to distinguish between subtypes of pericytes by differential co-staining with multiple pericyte markers.

## Pericyte regulation of vascular homeostasis

Pericytes are vital to vascular stability and to the regulation of vascular structure. Many reports show that the different subtypes of pericytes have different effects on the structure and function of blood vessels. It appears that PDGFR-β-type pericytes are derived from MSCs and differentiate into smooth muscle cells.^26,29,30^ PDGFRβ serves as a major pericyte marker and is involved in neurovascular functions, neuronal development, and aging.^31–33^ It regulates the development of cerebral microcirculation and the blood–brain barrier (BBB) and is associated with vascular disorders of the CNS.^32,34,35^ CD146 is an endothelial biomarker as well as a pericyte marker.^13,35^ CD146 type pericytes can be derived from CD146^+^ cells in bone marrow aspirates,^36^ or from neural precursor cells.^33^ Deletion of CD146 in pericyte caused an impairment in pericyte recruitment and the breakdown of BBB.^33,37^ Interestingly, CD146 coregulates with PDGFRβ in pericytes, which is required for pericyte recruitment, BBB development, and formation.^33,37^ It was revealed that the dimerization of CD146 is required for its association with PDGFRβ, which facilitate the auto-phosphorylation of PDGFRβ, the recruitment of PI3K-p110 to the p-PDGFRβ, and the downstream signaling of PDGFRβ, leading to pericyte recruitment and BBB integrity.^33,37^ CD146 also appears to be an orchestrator of the vascular niche that is involved in ischemic and atherosclerotic heart conditions.^15,38,39^ Desmin-type pericytes are derived from hematopoietic precursor cells and differentiate into fibrocytes.^40,41^ Like other pericyte markers, desmin is also involved in the atherosclerotic process via its regulation of stem cells and endothelial cells.^42,43^ NG2^+^ cells were identified in glia of the central nervous system (CNS),^44^ and NG2 is detected in arteriolar pericytes but not venular pericytes in the mesenteric microvascular system.^45,46^ In addition, NG2 is expressed in arterioles, capillaries, and venules pericytes in the retinal microvascular segments.^47^ NG2-type pericytes can also be derived from adipose-derived stem cells and differentiate into specialized adipose cells.^48,49^ Collectively, pericytes play a critical role in the formation and repair of the structural integrity of blood vessels. Pericyte deficiency or pathological alterations in pericytes could lead to dilated and hemorrhagic blood vessels, and is associated with disease processes.^50^

## Deficiency of existing classification

To date, a single specific marker that could be used solely to label pericytes is undefined. The expression of pericyte makers varies between the different stages of pericyte development, and according to various pathological processes as well as in the cell culture environment. Therefore, the effective and accurate identification of pericytes is an important issue to be resolved. At present, pericytes are distinguished by a means of a combination of morphology, the relationship to endothelial cells, and labeling by greater than two pericyte markers. These criteria provide a basis by which to promote further research on the classification of pericyte subtypes.

In addition, different names of pericytes have been used and the controversies of pericyte identity remain to be fully addressed including specific versus non-specific markers. Herein, we have summarized some commonly described names and their putative biomarkers in neural and spinal cord as well as in bone systems (Table 1). Further, we have provided an update on various Cre lines generated and their usefulness for pericyte studies relevant to SCI and bone (Table 2).^51–75^

**Table 1:** Classifications and biomarkers of perivascular stem cells

Classifications	Biomarkers	PDGFRβ	αSMA	CD146	desmin	NG2	CD13	CD44	VIM	Nestin	CD56	CD34	CD31	vWF	Sca-1	References
	Cell surface	+		+		+	+	+			+	+	+			
	Uniprot no. for human	P09619	P62736	P43121	P17661	Q6UVK1	P15144	P16070	P08670	P48681	P13591	P28906	P16284	P04275		
	Uniprot no. for mouse	P05622	P62737	Q8R2Y2	P31001	Q8VHY0	P97449	P15379	P20152	Q6P5H2	P13595	Q64314	Q08481	Q8CIZ8	P05533	
	Names descirbed (nomenclature)															
Perivascular cells at different locations	Pericytes in general	+	+	+		+		+		+	-	-	-	-		
	Pericytes in brain tissue	+	+	+	+	+	+									^10^
	Capillary-associated pericytes.	+	-	+		+	+									^10,52^
	Venules-associated pericytes		+			-										^52^
	Arterioles-associated pericytes		+			+										^13,46,51^
	Pericytes of microvessels		+													^52^
Perivascular cells at different stages	Perivascular cells		+			+										^52^
	Intermediate perivascular cells (PVC)		+			+										^52^
	Smooth muscle perivascular cells		+			-										^52^
	Bone marrow-derived pericyte precursors	+													+	^55^
	Attaching pericytes	+	+/−		+/−	+/−										^55^
	Mature pericytes	+/−	+/−		+/−	+/−										^55^
Pericytic markers related cells or structure	Mural cells in all large vessels		+			+										^52^
	Arteriole vSMC	+/−	+		+/−											^55^
	vSMCs in large vessels		+	+	+	+	+									^10^
	Mesenchymal stem cells			+				+								^52,54^
	Wharton’s jelly		+			-										^52^
Associated cells with no pericytic markers	Endothelial cells		-			-						+	+	+		^52,94^
	Adventitial cells		-									+			+	^52,56^
	Adventitial perivascular cells											+				^52^
Other species (Chiken) pericytes	Pericytes				+				+							^53^
	Smooth muscle perivascular cells		+		+				+							^53^

**Table 2:** Cre mouse lines and their usefulness for pericyte studies

Cre mouse lines	Targets	Their usefulness for pericyte studies	References
*In neural and SCI system*			
Wnt1-Cre, Sox10-Cre mice crossed to Rosa26(eYfp)	Tagging of Neural crest-derived MSC	Neural crest-derived perivascular cells	^61^
Foxg1(cre/+);Tgfbr2(flox/flox) (Tgfbr2-cKO)	Ko of Tgfbr2	Brain vessel development	^64^
Glial fibrillary acidic protein (GFAP)-cre	Ko of beta1-integrin (beta1-itg)	Perivascular astrocyte, blood–brain barrier	^71^
Nestin-Cre	Ko of CLEC-2	Maturation and integrity of the developing vasculature	^67^
Wnt1-cre	Ko of Ctnnb1 (beta-catenin)	Pituitary vasculature, neural crest-derived pericytes	^60^
NG2-CreERT:R26R-tdTomato	Ko of Ninj1 gene	Schwann cells and microvasculature	^69^
Zeb2 (Sip1/Zfhx1b) Ko with Tie2-Cre and Vav-iCre	Ko of Zeb2 (Sip1/Zfhx1b)	Pericyte coverage of the cephalic vasculature	^63^
R26R with SM22alpha-Cre	Ko of BMPR1A or MMP2	Brain microvessels	^41^
*In skeletal system*			
Tie2-Cre mice with R26Rosa-lox-Stop-lox-LacZ	Tagging of endothelial cells	Endothelial cells	^73^
Ocn-Cre, Dmp1-Cre and Cxcl12(gfp)	Tagging Cxcl12(gfp) expressing cells	Cxcl12-abundant reticular cells and arteriolar pericytes	^74^
Rosa26-YFP-Sox10-Cre	Tagging of YFP-positive PCs and vSMCs	PCs, vascular smooth muscle cells (vSMC)	^70^
Rosa26R-LacZ	Osterix-expressing osteoblast precursors	Coupled vascular and osteogenic transformation	^68^
Wnt1-Cre-Tom and GLAST-CreERT2-Tom	GLAST^+^ Wnt1-traced pericytes	Bone marrow pericytes	^75^
Msx1(lacZ), Msx2(lox) and Sm22alpha-Cre	Ko of Msx1(CreERT2)	Vascular smooth muscle cells (vSMC) in arteries	^66^
PDGFRalpha or PDGFRbeta Ko with SM22alpha-Cre	Ko of PDGFRalpha or PDGFRbeta	Vascular smooth muscle cells (vSMC) development	^62^
Bmp2 floxed/3.6Col1a1-Cre [Bmp2-cKO(od)]	Ko of Bmp2 gene in odontoblasts	Odontoblasts on vascular bed and associated pericytes	^72^
Alpha-SMA-GFP transgenic	Bone marrow stromal cells	Vascularization in bone microenvironment	^58^
*Other pericyte-related systems*			
Myh11-Cre(ERT2)tdTomato	Ko of KLF4	Smooth muscle cells or pericytes in adipose tissue	^57^
LysM-Cre	Ko of NG2	Tumor vascularization	^59^
Foxd1-Cre; Rs26-tdTomato	Foxd1 progenitor-derived pericytes	Myofibroblast precursor	^65^

## The role of pericytes in SCI

SCI is a devastating and traumatic insult to the spinal nervous system, which is a population-health burden by the characteristics of high incidence, high morbidity, high cost, and presents a significant challenge for patients, care workers, and clinicians.^76^ The microcirculatory disturbance that occurs during the early stage of SCI causes localized edema, ischemia, and hypoxia, which leads to secondary injury, including anaerobic metabolism, tissue acidosis, free radical reaction, ion pump disorders, and a series of biochemical reactions, which further exacerbates the deterioration of spinal cord tissue.^77,78^ Meanwhile, vascular lesions, inflammation, and other factors will result in the formation of a necrotic cavity, spinal cord tissue softening, and possibly the formation of a glial scar, leading to the permanent loss of the spinal cord function.^79,80^ Although several methods, such as surgical anastomosis and physical rehabilitation, have relieved the pathological changes of SCI by varying degrees, none of these methods has a curative clinical effect and the prognosis of SCI is poor.^81^

**Fig. 2:**
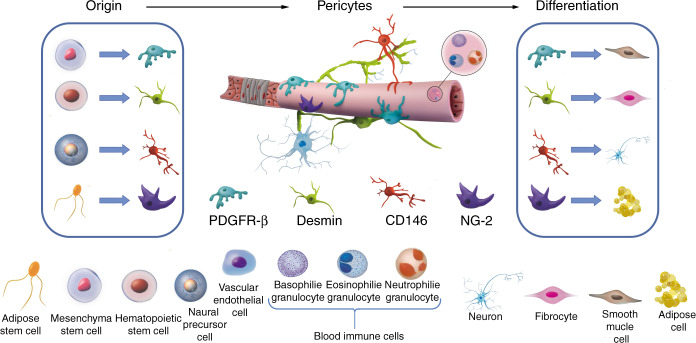
Schematic model showing pericytes by the expression of pericyte markers of PDGFRβ, CD146, desmin, and NG2 as examples, and their potential differentiation lineages

Cell transplantation has been proposed as a potential treatment of SCI. However, due to the damage of local blood vessels, lack of oxygen and nutrients, and the apoptotic death of neural cells, the survival of the transplanted cells for the treatment of SCI is jeopardized without the support of the local blood vasculature.^82^ Thus, vascular regeneration is an important and challenging topic of SCI treatment. The neurovascular unit (NVU) of the spinal cord consists of the spinal cord vascular endothelial cells, vascular basement membranes, pericytes, glial cells, and adjacent neurons. Coordinated intercellular communication among neighboring cells is required to maintain the functional and structural stability of the NVU, which is mediated by autocrine and paracrine signaling mechanisms. Pericytes are favorably positioned in the NVU between endothelial cells, neurons, and astrocytes, to facilitate the coordination of intercellular communication, such as pericyte-endothelial signaling and pericyte-astrocyte signaling, which are involved with the regulation of vital functions of the CNS. Pericyte-signaling systems can regulate the integrity of the BBB, angiogenesis, phagocytosis, cerebral blood flow (CBF), capillary diameter, neuro-inflammation, and multipotent stem cell activity.^35^ The NVU is possibly the most fundamental structure maintaining the blood spinal cord barrier in the spinal microenvironment.^83^ The pericyte is a fundamental cell of the NVU, and performs vital functions, including neuroprotection, maintaining the pluripotency of stem cells, regulation of the BBB, and the promotion of angiogenesis and vessel maturation.^84–86^ The role of pericytes in the regulation of CBF, neurovascular coupling, and neurodegenerative conditions remain debatable. To date, the pericyte has been found to play an important role in maintaining the blood spinal cord barrier and vascular regeneration.^86^ Following SCI, new blood vessels are required to restore blood supply to damaged areas, relieve localized hypoxia, and reduce nerve apoptosis. To repair damaged blood vessels, the number and location of pericytes must also be restored. At present, owing to the absence of specific markers, there is considerable ambiguity of the distribution, number, and function of different subtypes of pericytes. Several subtypes of pericytes might have the potential for neural cell differentiation and are able to contribute to tissue repair and regeneration of SCI (Fig. 2). Further, the proper arrangement among subtypes of pericyte could provide a novel therapeutic basis to promote angiogenesis and axon regeneration following SCI.

Pericytes are thought to regulate blood flow within the microvessels by constriction, and the flow of some particles in the blood vessel across the BBB, for the maintenance of the function of the nervous system and the integrity of the BBB.^29^ Without pericytes, large proteins can cross the spinal cord barrier by transcytosis, and affect the proliferation and differentiation of endothelial cells, leading to vascular malformations.^83,87^ Microvascular pericytes directly contact endothelial cells by gap junctions, thereby regulating endothelial cell proliferation and thus restricting vascular malformations.^88^ Accordingly, the pericyte is an important cell to help maintain the homeostasis of the vascular microenvironment following SCI. To date, relative to endothelial cells, our understanding of the roles and functions of pericytes is limited.

## The functions of the subtypes of pericytes in SCI

**Fig. 3:**
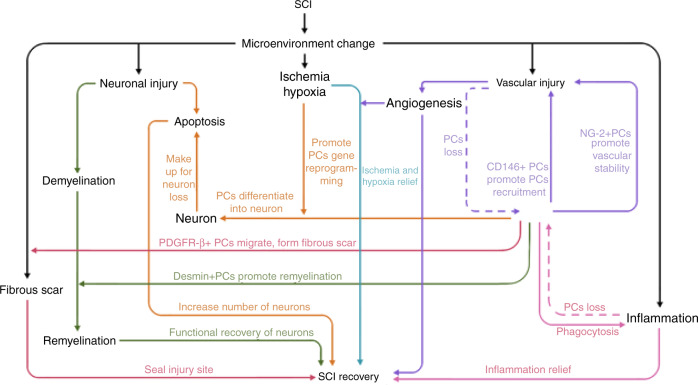
Relationships between different types of pericytes and the SCI microenvironment

The above common subtypes of pericytes participate in angiogenesis and functional protection from SCI. Previous research indicates that pericytes play an important role in several aspects of SCI (Fig. 3), including (1) nerve regeneration: CD146 type pericytes can differentiate into neurocyte and replace neurons in damaged areas; (2) microenvironment: the inhibition of pericyte contraction could effectively alleviate local tissue hypoxia after SCI;^89^ (3) post-SCI angiogenesis: NG2-type pericytes promote revascularization, vascular stability, and tissue healing;^90^ (4) fibrotic scar: PDGFR-β type pericytes are the source of the scar-forming cells and NG2-type pericytes promote scar formation.^91^ Although there are reports indicating that fibrotic scarring has a negative role in the repair of SCI, some pericyte-induced fibrotic scarring may have a positive effect on the recovery and tissue healing of SCI. Excessive inhibition or activation of certain types of pericytes can lead to an open tissue defect, whereas moderate pericyte proliferation contributes to wound closure and reduction of the fibrotic scar.^92^ Therefore, the pericyte is potentially a new target for the treatment of SCI. Determination of and promoting the therapeutic activation of the specific pericyte subtype ratio conducive to the recovery of SCI, represents a promising and challenging aim for future research. The promotion of angiogenesis following pericyte transplantation, and improvement of the microenvironment of the damaged zone during functional recovery are fundamental areas that must be solved for the application of pericyte-based treatments for SCI.

## Pericyte coverage of blood vessels in spinal cord

Pericytes are localized on the surface of the capillary, and play an important role in angiogenesis, vascular remodeling, and stability.^12,93^ Pericytes are relatively abundant in the spinal tissues as the coverage of pericytes in the spinal microvasculature is higher than that in the peripheral tissues. This phenomenon suggests that spinal cord pericytes may have a tissue-specific function. The decrease of pericyte coverage is accompanied by the decrease of vascular density in the peripheral tissues. The abnormal pathological changes of blood vessel characteristics, such as increased diameter, tortuosity, reduced perfusion, and depression may be the result of the changes that occur in the spinal cord microenvironment during the early stage of SCI.^30^ The coverage rate of pericytes is ~39.77% in ventral horn blood vessels and 13.34% in dorsal horn blood vessels.^94^ However, the proportion in the microvasculature of striated muscle is estimated at 1:100^29^. Our recent data also suggest that the locations and morphologies of common subtypes of pericyte vary in the normal spinal environment, as seen by immunofluorescence staining of spinal cord vessels and capillaries (unpublished observation). There appears a certain proportion and close physical connection among common subtypes of pericyte in the spinal cord. The size ratio of pericyte and vascular endothelial cells is ~1:2. In other words, the size of a pericyte is approximately equal to that of two vascular endothelial cells.^29,30^ This quantitative ratio might help us determine the total number of pericytes based on the number of endothelial cells in pericyte transplantation. By using the optimal number of transplanted cells with subtypes of pericytes, the goal of restoring the microenvironment of the SCI and recovering the function of the injured spinal cord can be achieved.

## Application potential of pericytes in neurologic diseases and SCI repair

At present, there are two potential methods for treating diseases using the stem cell potential of pericytes: (1) exogenous pericyte cell transplantation, and (2) mobilization of endogenous pericyte cell migration. Current research mainly uses exogenous pericyte transplantation to repair tissue damage, and the endogenous pericytes mobilization method is still in the theoretical stage.^95^ Most pericyte transplantation mediates therapeutic effects by the secretory mechanism of pericytes to repair damaged nerve tissue. For instance, the therapeutic effects and mechanistic targets of saphenous vein-derived pericyte progenitor cells were investigated in an infarcted heart model, and found that the delivered cells could signal through a paracrine mechanism to reduce myocardial scarring, apoptosis, and fibrosis, while increasing vascular stability and attenuating permeability.^96^ Pericytes can promote the activation of stationary satellite cells by secreting insulin-like growth factor-1 or Ang1, and activated satellite cells may subsequently differentiate into myoblasts with tissue regenerative potential.^95^

Further, the repair and reconstruction of vascular tissues also depend on post-natal adult MSCs. There are many similarities between pericytes and stem cells. The pericyte is a source of precursor cell which has broad potential therapeutic applications and is an important member of the stem cell family, and has the capabilities of self-renewal, multi-directional differentiation, and immune suppression.^29,30^

Research has revealed that pericytes play a significant role by enhancing the structural integrity and functionality of the new blood vessels, particularly for the regulation of neurovascular function.^19^ MSC-derived pericytes appear to have potential in the treatment for Alzheimer’s disease by their ability to decrease amyloid-β-related pathology through protease-mediated degradation and to restore cerebral circulation.^97^ Consistently, pericytes appear to have an established protective role against neuronal damage in neurodegenerative diseases, via promoting a less aggressive neuronal environment and regulating neurovascular function in the CNS.^98,99^ These findings suggest that pericytes play a significant role in vascular regeneration and the regulation of neurovascular protection in the CNS. The widespread distribution of pericytes provides an adequate source of cells for the clinical application of autologous pericytes. At present, specific identification and isolation of pericytes have not been well established and the clinical application of pericytes is limited.

The microcirculatory disturbance in the early stage of SCI causes localized edema, ischemia, and hypoxia, leading to secondary injury including anaerobic metabolism, tissue acidosis, free radical reaction, and ion pump failure. This series of reactions give rise to ischemia and hypoxia in the spinal cord microenvironment, resulting in apoptosis and phagocytosis of spinal cord cells.^100^ The failure of endogenous angiogenesis directly limits the therapeutic effect of SCI treatments because new blood vessels are deprived of necessary and appropriate subsets of pericytes.^93,101^ Therefore, the pericyte has become a new target for SCI repair. Through gaining the knowledge and understanding of neuro-pathophysiology and the function of pericytes, the single subtype of pericyte transplantation appears to be the most common treatment of SCI and vascular regeneration.

The transplantation of pericytes may be ineffective owing to the low survival rate of transplanted pericytes in the transplant site where there is a lack of vascular support.^102^ The survival rate of transplanted pericytes has been shown to be independent of the cell density, and >80% of pericytes might die after transplantation for SCI.^103^ Therefore, methods to promote the survival and differentiation of the transplanted pericytes in the SCI microenvironment remains a challenge for the clinical application of pericyte-based therapies. At present, the survival rate of pericyte cell transplantation might be improved by combining pericytes with biological materials for delivery. For example, pericytes transplantation may be applied using a tissue scaffold for the treatment of spinal disease of the lumbar vertebra, and transplantation of a collagen matrix containing pericytes and endothelial cells may be effective for the promotion of angiogenesis during epidermal tissue regeneration.^95^

## Controversy of pericytes in the formation of scarring of SCI

Contrary to the potential beneficial effect on SCI, pericytes were found to promote the formation of fibrotic scars and worsen spinal cord injuries.^104^ For instance, the number of new pericytes after SCI is twice that of astrocytes, whereas there are ten times as many astrocytes as pericytes in normal spinal tissue.^26^ These findings indicate that the PDGFR-β^+^ pericytes are a source of scar-forming cells in spinal cord lesions and inhibit axon regeneration in the adult spinal cord.^26^ Research also indicates that inhibiting the proliferation of the PDGFR-β^+^ pericytes reduces fibrotic scar formation by fibroblasts that are derived from pericytes, which promotes axon regeneration and functional recovery following SCI.^92^ Both studies used the *Glast* promoter-based transgene to select pericytes. However, the *Glast* gene is also expressed in other cell types, including astrocytes, Bergmann glia cells, Muller cells, and neural stem cells.^105–108^ Therefore, use of the *Glast* promoter-based transgene appears to be inconclusive and warrants further investigation. Immunofluorescence imaging shows that astrocytes have a greater basement membrane association as compared to the PDGFR-β^+^ pericytes.^26,91^ Furthermore, it appears that the mice in which the pericyte function is impaired, might sustain further CNS damage because of open SCI lesions. Moreover, pericytes are important for the formation of the BBB and vascular network, and removing pericytes from the damaged area might result in brain damage.^91^ The fibrotic scarring may help to isolate the inflammatory areas to maintain the integrity of the nerve tissue.^91^ Other evidence indicates that PDGFR-β^+^ pericytes play a positive role in sealing the lesion core following SCI, akin to the well-established protective role of reactive astrocytes.^108^ Recent research shows that the scar formation by pericytes occurs at the center of the injury, while scar formation by astrocytes is located at the periphery of the injury, indicating that when pericytes fail to proliferate, an open tissue defect may develop.^109^

## The role of pericytes in bone development and fracture repair

Vascular regeneration is essential in bone development and repair, which is mediated by endothelial cells and pericytes.^110–112^ Endothelial cells are major components of the vasculature and form a crucial network between the bone system and blood circulation system. Pericytes, also known as mural cells, are perivascular cells surrounding blood vessel capillaries and endothelial cells, which facilitate the formation, maturation, and maintenance of the vascular network in bone.^113–115^ Pericytes also act as a stem cell source that is important for bone tissue regeneration, with several studies demonstrating their osteogenic potential.^16,116–118^ Recent research has focused on defining biomarkers that typify pericytes with the greatest potential for osteoblast formation.^16^ Importantly, CD146^+^ pericytes derived from skeletal tissue appear to have the most potential for osteoblast formation in vitro and skeletogenesis in vivo in comparison to pericytes derived from soft tissues, such as adipose or dermal tissue, suggesting the tissue-specific function of various pericyte populations.^16^ Nonetheless, pericytes derived from soft-tissue sources, such as adipose, have also shown a tremendous capacity for bone regeneration in clinical models.^7,119–127^

The role of perivascular progenitor cells in bone development and repair is emerging in vitro and in vivo. Early studies have shown that Gli-1-positive perivascular cells act as an innate osteogenic potential of perivascular progenitors involved in ischemia-induced vascular remodeling.^14,128^ During the process of embryonic endochondral ossification, Osx1 positive osteoprogenitor cells from limb mesenchyme with attachment to the blood vessels were able to invade the cartilaginous anlagen of long bones, indicating a role of MSCs-like cells, in bone development.^129^ In addition, endothelial and perivascular cells labeled with intravascular dyes were tracked to be present within new bone and cartilage in animal studies.^130–132^ Further, using lineage-tracing experiments, SMA tracked by an inducible reporter animal was found to act like osteochondroprogenitor cells.^133^ In this study, inducible SMA reporter mice were used to show that SMA-expressing cells were present in long bone fracture callus.^133^ Although SMA is not a definitive marker of pericytes (as it was also expressed in other cell types such as osteoblasts precursor cells, smooth muscle cells, and myofibroblasts), it is suggested that pericyte like cells play an important role in the early development of the skeletal system.^134^

More recently, pericytes have been identified as a cell source with osteogenic properties for bone fracture repair.^118^ In this study, pericytes were isolated from mouse embryos at 14.5–16.5 days post-coitus using CD146 as a cell surface marker. An enriched homogenous population of pericytes was also isolated by using dual markers of CD146 and NG2 with a lack of expression of CD31, CD45, and Ter119. These pericytes were also found to express high levels of PDGFRβ, which is not expressed in MSCs. Interestingly, all NG2, CD146, and PDGFRβ-positive pericytes showed osteogenic, adipogenic, and chondrogenic differentiation ability. Further, lineage-tracing experiments using the NG2-Cre or tamoxifen-inducible NG2-CreER mouse line showed that pericytes could differentiate into osteogenic cells in mice.^118^ In a bone fracture model, pericytes labeled with tdTomato-expressing were found to be recruited to the newly developed callus of the fractured femurs, which showed positive expression of Runx2 and type I collagen, in line with observed improved bone fracture repair.^118^

The therapeutic potential of pericytes in non-union fracture was also investigated using a rat tibial atrophic non-union model, in which pericytes were injected by a percutaneous route 3 weeks after the fibrous non-union procedures.^125^ It was revealed that injection of pericytes increased fracture callus size, accompanied by mineralization and osseous union. Taken together, it is likely that local injection of pericytes is useful for the repair of a delayed union or non-union bone fracture.^125^

## The role of pericytes in bone formation and defect healing

The skeletal regenerative potential of exogenous PSCs derived from adipose tissue has been reported.^121^ The bone-forming capacity of purified PSCs was investigated using a murine calvaria defect model of bone healing.^121^ In this study, PSCs were isolated from a patient and implanted in a 3 mm non-healing calvaria defect centered in the parietal bone. Radiographic and histologic analysis showed that PSCs led to a significant increase in bony regeneration at the defect site with significant bone defect healing over time. In comparison, the unpurified stromal vascular fraction from the same patient had no statistically significant benefit in comparison to an acellular scaffold control.^121^ PSCs appear to have the potential to prevent atrophic non-union during fracture healing and could provide therapeutic benefit for a developing non-union.^125^

Consistently, ectopic bone formation has been demonstrated in implanted pericytes.^126^ For example, recent research showed that pericytes derived from human white adipose tissue with positive expression of alkaline phosphatase and bone matrix have osteogenic potential in an intramuscular mouse model.^126^ Further, PSCs have synergistic effects on ectopic bone formation when jointly used with bone morphogenetic protein 2 (BMP2).^7^ Similarly, in heterologous xenograft models, CD146^+^ pericytes derived from human adipose tissue were transplanted into animals and found to promote bone formation.^135^ In contrast, CD31^+^ endothelial cells were shown to suppress osteogenic differentiation.^136,137^

Collectively, pericytes or PSCs showed significantly greater potential for bone formation in comparison with unpurified stroma in both ectopic and orthotopic bone models. However, whether PSCs might induce bone defect healing through direct ossification of PSCs or indirect paracrine effects exerted by PSCs, or via the elimination of an inhibitory cell type within the heterogeneous stroma, remains to be elucidated. Further research is required to enrich tissue-specific PSCs that are optimized for skeletal tissue regeneration.

## The role of pericytes in osteonecrosis

Pericytes could be an optimal source of stem cells in the treatment of osteonecrosis (avascular necrosis), the in situ death of a segment of bone, often affecting the femur or humerus.^138,139^ Although the loss or reduction of blood supply is necessary for osteonecrosis to occur, the pathogenesis of osteonecrosis remains incompletely understood.^138,140^ Osteonecrosis frequently affects younger patients aged 30–40 years, whereas its onset and progression are unpredictable.^138,141^ Osteonecrosis might be a disease initiated at a cellular level from bone marrow stromal cells and, therefore, stem cell therapy represents a promising treatment strategy.^140,142^ A recent systematic review and meta-analysis found that early autologous MSC implantation could attenuate disease progression, thereby alleviating the need for joint surgery.^140^ Additional studies suggest the potential benefit of stem cell therapy for the treatment of osteonecrosis.^143–145^ Despite the supporting evidence, further research is required to improve the effectiveness of stem cell therapy for osteonecrosis, specifically relating to optimal cell sources, number, and translation.^144^ Purified MSC-like pericytes (CD146^+^, CD34^−^, CD45^−^) represent a promising population in orthopedics and tissue engineering owing to their osteogenic, angiogenic, and paracrine activity for the secretion of vital growth and differentiation factors.^110,126^ Very little is known about the role of pericytes in osteonecrosis pathogenesis or treatment. Pericytes could be important cells for regulating the balance of physiological bone remodeling, with a loss of pericyte function implicated in osteonecrosis progression.^146^ Future research is necessary to further characterize tissue-specific pericytes that could both illuminate the pathogenesis of osteonecrosis and be an optimal stem cell source for therapeutic applications of osteonecrosis.

## The role of pericytes in spinal fusion

Spinal fusion has become a major orthopedic procedure with a need for improved efficacy of various fusion techniques for various indications.^147^ Interestingly, PSCs were found to facilitate bone formation in a spinal fusion model and could potentially be applied for tissue regeneration using technologies, such as bone graft substitute or scaffold.^124^ Specifically, human PSCs were seeded in a demineralized bone matrix scaffold and tested in a rat posterolateral lumbar spinal fusion model, and found to increase endochondral ossification, bone deposition, and bone strength.^124^ Human PSCs were also found to have greater bone-forming potential in a posterolateral lumbar spinal fusion,^124^ as compared with the acellular control group in an athymic rat model. Using species-specific immunohistochemistry, PSCs were present within and around the newly formed bone tissue.^124^ Further, in a rat model of gonadectomy-induced osteoporosis, implanted PSCs showed protective effects on systemic osteoporosis.^120^ When PSCs were applied to a spinal fusion model in rats that had osteoporotic pathology induced ovariectomy, they showed a protective effect on bone-forming ability with a higher density of PSCs required.^120^

Despite recent progress showing the potential of pericytes in bone development, bone fracture, and bone defect repair, the isolation or purification of pericytes is still changing as many markers used are not pericyte-specific. For instance, CD146 used for pericytes is also expressed in MSCs,^148^ whereas NG2 used for pericytes is not expressed in MSCs,^13,149^ resulting in different subsets of PSCs. It remains a challenge to isolate and culture PSCs by avoiding the biologic property shift and regulatory measurements under cell culture conditions. Further, there were several subtypes of pericytes with different biomarkers. It will be important to use more specific markers to directly identify and isolate each specific subtype of perivascular progenitors, and to compare them individually and in combination for their therapeutic benefits in bone formation and tissue regeneration.

## Future direction: proportional arrangements of pericytes for SCI repair and bone repair

It is speculated that the proportional arrangement of common subtypes of pericyte, such as PDGFR-β type, Desmin type, CD146 type, and NG2 type is of great significance for the stability of the spinal cord microenvironment. For instance, the PDGFR-β type pericytes may have the potential to close and seal lesions, thus preventing further damage to the CNS;^91,150^ the CD146 type pericyte may be able to secrete cell adhesion molecules and help pericytes to attach to endothelial cells;^151^ the NG2 type pericytes could possibly promote angiogenesis and vascular structural stability.^90^ Reports indicate that the different types of pericyte have different functions and that the intercellular communication among subtypes of pericyte may be of great significance, however, the specific mechanisms have not been clearly elucidated.^29,90,91^ Hypothetically, when the ratio of these pericytes is abnormal, CNS dysfunction might adversely occur. Similarly, it is postulated that different arrangements of pericytes will delay or facilitate bone repair. Further quantitative assessment on the proportional arrangements of subtypes of pericytes in a tissue-specific manner, will help to elucidate their approximate ratio for optimal vascular network and tissue regeneration. Collectively, we have highlighted common pericyte subtypes and their progenitors, and introduced a novel concept of the importance of maintaining proper pericyte subtype ratios in SCI and bone repair. This concept provides a theoretical basis for a potential new treatment method for SCI and bone fracture or defect, aiming to restore the number and proportion of the common pericyte subtypes through pericyte transplantation.

## Conclusions

**Fig. 4:**
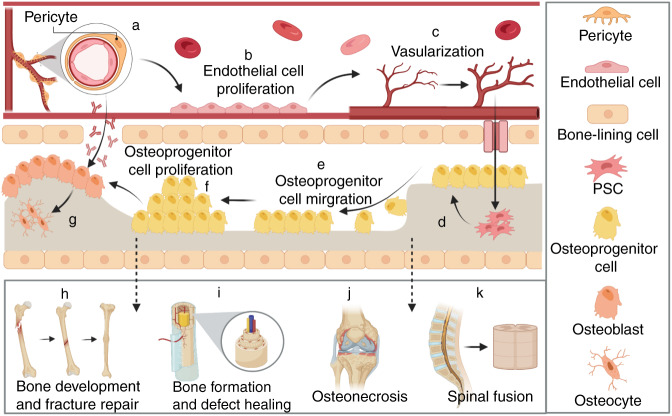
Schematic diagram proposing the role of pericytes in angiogenesis and bone repair. **a** Pericytes, located in the basement membrane of blood vessels, are the cells that surround endothelial cells in the capillary and veins of the body. **b** Pericytes communicate with endothelial cells through physical contact and paracrine signals, monitoring and stabilizing the maturation of endothelial cells. **c** Pericytes promote angiogenesis, which has an important role in maintaining intravascular homeostasis. **d**–**k** Pericytes are also an important source of stem cells for bone regeneration with osteogenic potential. **d** Mediating the differentiation of perivascular stem cells (PSCs) into osteoprogenitor cells. **e** Promoting the migration of osteoprogenitor cells. **f** Inducing the proliferation of osteoprogenitor cells. **g** Inducing the differentiation of osteoblasts via paracrine mode. **h**–**k** The proposed role of PSCs in bone development and fracture repair (**h**), bone formation and defect healing (**i**), osteonecrosis (**j**), and spinal fusion (**k**) as discussed in the manuscript

We summarize the current knowledge concerning the roles of common subtypes of pericytes in relation to the repair of SCI and bone defects. The heterogeneity in pericyte marker expression reflects the heterogeneity in function and differentiation potential of these cells. Advanced techniques are to be further employed to explore the function of pericytes in SCI, and to dissect the role of pericytes in scarring, angiogenesis, and axon regeneration. We have further outlined the lineages and differentiation paths of pericyte subtypes. We have raised an interesting and novel idea that optimal proportional ratios of different pericyte subtypes exist in spinal cord tissue, and thus may play a role in SCI recovery. Pericytes are also key components of the basic multicellular unit including endothelial cells, osteoclasts, osteoblasts, osteocytes, and bone lining cells of the skeletal system (Fig. 4). Understanding the role of pericytes in the bone microenvironment may help to develop novel therapeutic targets and diagnostic biomarkers for bone diseases, such as osteoporosis, osteonecrosis, osteoarthritis, and delayed fracture healing. Further unraveling the subtypes and functions of pericytes, developing approaches for pericyte isolation using specific cell surface markers, as well as the quantitative relation among subtypes are urgent issues to be solved in the potential clinical application of pericytes.

## References

[1] Armulik A, Genove G, Betsholtz C (2011). Pericytes: developmental, physiological, and pathological perspectives, problems, and promises. Dev. Cell.

[2] Armulik A (2010). Pericytes regulate the blood-brain barrier. Nature.

[3] Dalkara T, Gursoy-Ozdemir Y, Yemisci M (2011). Brain microvascular pericytes in health and disease. Acta Neuropathol..

[4] Winkler EA (2013). Blood-spinal cord barrier breakdown and pericyte reductions in amyotrophic lateral sclerosis. Acta Neuropathol..

[5] Zhou W (2017). Targeting glioma stem cell-derived pericytes disrupts the blood-tumor barrier and improves chemotherapeutic efficacy. Cell Stem Cell.

[6] Oudega M (2012). Molecular and cellular mechanisms underlying the role of blood vessels in spinal cord injury and repair. Cell Tissue Res..

[7] James AW (2012). Perivascular stem cells: a prospectively purified mesenchymal stem cell population for bone tissue engineering. Stem Cells Transl. Med..

[8] Paquet-Fifield S (2009). A role for pericytes as microenvironmental regulators of human skin tissue regeneration. J. Clin. Investig..

[9] Yao Y, Chen ZL, Norris EH, Strickland S (2014). Astrocytic laminin regulates pericyte differentiation and maintains blood brain barrier integrity. Nat. Commun..

[10] Smyth LCD (2018). Markers for human brain pericytes and smooth muscle cells. J. Chem. Neuroanat..

[11] Nakagomi T, Nakano-Doi A, Kawamura M, Matsuyama T (2015). Do vascular pericytes contribute to neurovasculogenesis in the central nervous system as multipotent vascular stem cells?. Stem Cells Dev..

[12] Collett GD, Canfield AE (2005). Angiogenesis and pericytes in the initiation of ectopic calcification. Circ. Res..

[13] Crisan M (2008). A perivascular origin for mesenchymal stem cells in multiple human organs. Cell Stem Cell.

[14] Kramann R (2016). Adventitial MSC-like cells are progenitors of vascular smooth muscle cells and drive vascular calcification in chronic kidney disease. Cell Stem Cell.

[15] Chen WC (2015). Human myocardial pericytes: multipotent mesodermal precursors exhibiting cardiac specificity. Stem Cells.

[16] Xu J (2020). Comparison of skeletal and soft tissue pericytes identifies CXCR4^+^ bone forming mural cells in human tissues. Bone Res..

[17] Dellavalle A (2007). Pericytes of human skeletal muscle are myogenic precursors distinct from satellite cells. Nat. Cell Biol..

[18] Bice BD (2017). Environmental enrichment induces pericyte and IgA-dependent wound repair and lifespan extension in a colon tumor model. Cell Rep..

[19] Cheng L (2013). Glioblastoma stem cells generate vascular pericytes to support vessel function and tumor growth. Cell.

[20] Yamauchi M, Barker TH, Gibbons DL, Kurie JM (2018). The fibrotic tumor stroma. J. Clin. Investig..

[21] Birbrair A (2017). How plastic are pericytes?. Stem Cells Dev..

[22] Guimaraes-Camboa N (2017). Pericytes of multiple organs do not behave as mesenchymal stem cells in vivo. Cell Stem Cell.

[23] Xu J, Nie X, Cai X, Cai CL, Xu PX (2014). Tbx18 is essential for normal development of vasculature network and glomerular mesangium in the mammalian kidney. Dev. Biol..

[24] Lyle LT (2016). Alterations in pericyte subpopulations are associated with elevated blood-tumor barrier permeability in experimental brain metastasis of breast cancer. Clin. Cancer Res..

[25] Farrington-Rock C (2004). Chondrogenic and adipogenic potential of microvascular pericytes. Circulation.

[26] Göritz C (2011). A pericyte origin of spinal cord scar tissue. Science.

[27] Birbrair A (2014). Type-1 pericytes accumulate after tissue injury and produce collagen in an organ-dependent manner. Stem Cell Res Ther..

[28] Birbrair A (2014). Type-2 pericytes participate in normal and tumoral angiogenesis. Am. J. Physiol. Cell Physiol..

[29] Peppiatt CM, Howarth C, Mobbs P, Attwell D (2006). Bidirectional control of CNS capillary diameter by pericytes. Nature.

[30] Yemisci M (2009). Pericyte contraction induced by oxidative-nitrative stress impairs capillary reflow despite successful opening of an occluded cerebral artery. Nat. Med..

[31] Daneman R, Zhou L, Kebede AA, Barres BA (2010). Pericytes are required for blood-brain barrier integrity during embryogenesis. Nature.

[32] Bell RD (2010). Pericytes control key neurovascular functions and neuronal phenotype in the adult brain and during brain aging. Neuron.

[33] Chen J (2017). CD146 coordinates brain endothelial cell-pericyte communication for blood-brain barrier development. Proc. Natl. Acad. Sci. USA.

[34] Winkler EA, Bell RD, Zlokovic BV (2010). Pericyte-specific expression of PDGF beta receptor in mouse models with normal and deficient PDGF beta receptor signaling. Mol. Neurodegener..

[35] Sweeney MD, Ayyadurai S, Zlokovic BV (2016). Pericytes of the neurovascular unit: key functions and signaling pathways. Nat. Neurosci..

[36] Hassanpour M (2020). Autophagy modulation altered differentiation capacity of CD146^+^ cells toward endothelial cells, pericytes, and cardiomyocytes. Stem Cell Res. Ther..

[37] Chen J (2018). CD146 is essential for PDGFRbeta-induced pericyte recruitment. Protein Cell.

[38] Chen CW (2013). Human pericytes for ischemic heart repair. Stem Cells.

[39] Wilkinson FL (2007). Contribution of VCAF-positive cells to neovascularization and calcification in atherosclerotic plaque development. J. Pathol..

[40] Nehls V, Denzer K, Drenckhahn D (1992). Pericyte involvement in capillary sprouting during angiogenesis in situ. Cell Tissue Res..

[41] El-Bizri N (2008). SM22alpha-targeted deletion of bone morphogenetic protein receptor 1A in mice impairs cardiac and vascular development, and influences organogenesis. Development.

[42] Bruijn LE, van den Akker B, van Rhijn CM, Hamming JF, Lindeman JHN (2020). Extreme diversity of the human vascular mesenchymal cell landscape. J. Am. Heart Assoc..

[43] Kelly-Goss MR, Sweat RS, Stapor PC, Peirce SM, Murfee WL (2014). Targeting pericytes for angiogenic therapies. Microcirculation.

[44] Binamé F (2014). Transduction of extracellular cues into cell polarity: the role of the transmembrane proteoglycan NG2. Mol. Neurobiol..

[45] Alon R, Nourshargh S (2013). Learning in motion: pericytes instruct migrating innate leukocytes. Nat. Immunol..

[46] Murfee WL, Skalak TC, Peirce SM (2005). Differential arterial/venous expression of NG2 proteoglycan in perivascular cells along microvessels: identifying a venule-specific phenotype. Microcirculation.

[47] Chan-Ling T, Hughes S (2005). NG2 can be used to identify arteries versus veins enabling the characterization of the different functional roles of arterioles and venules during microvascular network growth and remodeling. Microcirculation.

[48] Volz, K. S. et al. Pericytes are progenitors for coronary artery smooth muscle. *eLife* **4**, e10036 (2015). 10.7554/eLife.10036 PMC4728130 26479710

[49] Ivanova E, Kovacs-Oller T, Sagdullaev BT (2017). Vascular pericyte impairment and connexin43 gap junction deficit contribute to vasomotor decline in diabetic retinopathy. J. Neurosci..

[50] Ahmed TA, El-Badri N (2018). Pericytes: the role of multipotent stem cells in vascular maintenance and regenerative medicine. Adv. Exp. Med. Biol..

[51] Crisan M (2009). Perivascular multipotent progenitor cells in human organs. Ann. N. Y. Acad. Sci..

[52] Crisan M, Corselli M, Chen WC, Peault B (2012). Perivascular cells for regenerative medicine. J. Cell Mol. Med..

[53] Fujimoto T, Singer SJ (1987). Immunocytochemical studies of desmin and vimentin in pericapillary cells of chicken. J. Histochem. Cytochem..

[54] Gronthos S, Simmons PJ, Graves SE, Robey PG (2001). Integrin-mediated interactions between human bone marrow stromal precursor cells and the extracellular matrix. Bone.

[55] Hall AP, Westwood FR, Wadsworth PF (2006). Review of the effects of anti-angiogenic compounds on the epiphyseal growth plate. Toxicol. Pathol..

[56] Passman JN (2008). A sonic hedgehog signaling domain in the arterial adventitia supports resident Sca1^+^ smooth muscle progenitor cells. Proc. Natl. Acad. Sci. USA.

[57] Bulut GB (2021). KLF4 (Kruppel-Like Factor 4)-dependent perivascular plasticity contributes to adipose tissue inflammation. Arterioscler. Thromb. Vasc. Biol..

[58] Cai X (2009). Bone marrow derived pluripotent cells are pericytes which contribute to vascularization. Stem Cell Rev. Rep..

[59] Cejudo-Martin P, Kucharova K, Stallcup WB (2016). Role of NG2 proteoglycan in macrophage recruitment to brain tumors and sites of CNS demyelination. Trends Cell Mol. Biol..

[60] Davis SW (2016). beta-catenin is required in the neural crest and mesencephalon for pituitary gland organogenesis. BMC Dev. Biol..

[61] Foster K (2008). Contribution of neural crest-derived cells in the embryonic and adult thymus. J. Immunol..

[62] French WJ, Creemers EE, Tallquist MD (2008). Platelet-derived growth factor receptors direct vascular development independent of vascular smooth muscle cell function. Mol. Cell Biol..

[63] Goossens S (2011). The EMT regulator Zeb2/Sip1 is essential for murine embryonic hematopoietic stem/progenitor cell differentiation and mobilization. Blood.

[64] Hellbach N (2014). Neural deletion of Tgfbr2 impairs angiogenesis through an altered secretome. Hum. Mol. Genet..

[65] Hung C (2013). Role of lung pericytes and resident fibroblasts in the pathogenesis of pulmonary fibrosis. Am. J. Respir. Crit. Care Med..

[66] Lopes M (2011). Msx genes define a population of mural cell precursors required for head blood vessel maturation. Development.

[67] Lowe KL (2015). Podoplanin and CLEC-2 drive cerebrovascular patterning and integrity during development. Blood.

[68] Maes C (2010). Osteoblast precursors, but not mature osteoblasts, move into developing and fractured bones along with invading blood vessels. Dev. Cell.

[69] Tomita Y (2019). Ninjurin 1 mediates peripheral nerve regeneration through Schwann cell maturation of NG2-positive cells. Biochem. Biophys. Res. Commun..

[70] Trost A (2014). Characterization of dsRed2-positive cells in the doublecortin-dsRed2 transgenic adult rat retina. Histochem. Cell Biol..

[71] Venkatesan C, Birch D, Peng CY, Kessler JA (2015). Astrocytic beta1-integrin affects cellular composition of murine blood brain barrier in the cerebral cortex. Int J. Dev. Neurosci..

[72] Yang W (2012). Bmp2 is required for odontoblast differentiation and pulp vasculogenesis. J. Dent. Res..

[73] Zeisberg EM, Potenta S, Xie L, Zeisberg M, Kalluri R (2007). Discovery of endothelial to mesenchymal transition as a source for carcinoma-associated fibroblasts. Cancer Res..

[74] Zhang J, Link DC (2016). Targeting of mesenchymal stromal cells by Cre-recombinase transgenes commonly used to target osteoblast lineage cells. J. Bone Min. Res..

[75] Sierra R (2020). Contribution of neural crest and GLAST^+^ Wnt1^+^ bone marrow pericytes with liver fibrogenesis and/or regeneration. Liver Int..

[76] El Masri WS, Kumar N (2011). Traumatic spinal cord injuries. Lancet (Lond., Engl.).

[77] Fan B (2018). Microenvironment imbalance of spinal cord injury. Cell Transplant.

[78] Norden, D. M. et al. Bone marrow-derived monocytes drive the inflammatory microenvironment in local and remote regions after thoracic spinal cord injury. *J. Neurotrauma* **36**, 937–949 (2018). 10.1089/neu.2018.5806 PMC6484351 30014767

[79] Kumar N, Osman A, Chowdhury JR (2017). Traumatic spinal cord injuries. J. Clin. Orthop. Trauma.

[80] Taoka Y, Okajima K (1998). Spinal cord injury in the rat. Prog. Neurobiol..

[81] Silva NA, Sousa N, Reis RL, Salgado AJ (2014). From basics to clinical: a comprehensive review on spinal cord injury. Prog. Neurobiol..

[82] Hurtado A (2006). Poly (D,L-lactic acid) macroporous guidance scaffolds seeded with Schwann cells genetically modified to secrete a bi-functional neurotrophin implanted in the completely transected adult rat thoracic spinal cord. Biomaterials.

[83] Lee JY, Kim HS, Choi HY, Oh TH, Yune TY (2012). Fluoxetine inhibits matrix metalloprotease activation and prevents disruption of blood-spinal cord barrier after spinal cord injury. Brain.

[84] Kisler K (2017). Pericyte degeneration leads to neurovascular uncoupling and limits oxygen supply to brain. Nat. Neurosci..

[85] Ryu, B. et al. Allogeneic adipose-derived mesenchymal stem cell sheet that produces neurological improvement with angiogenesis and neurogenesis in a rat stroke model. *J. Neurosurg*. **132**, 442–455 (2019). 10.3171/2018.11.JNS182331 30797215

[86] Shaw I, Rider S, Mullins J, Hughes J, Peault B (2018). Pericytes in the renal vasculature: roles in health and disease. Nat. Rev. Nephrol..

[87] Bergers G, Song S, Meyer-Morse N, Bergsland E, Hanahan D (2003). Benefits of targeting both pericytes and endothelial cells in the tumor vasculature with kinase inhibitors. J. Clin. Investig..

[88] Alarcon-Martinez, L. et al. Capillary pericytes express alpha-smooth muscle actin, which requires prevention of filamentous-actin depolymerization for detection. *Elife* **7**, e34861 (2018). 10.7554/eLife.34861 PMC5862523 29561727

[89] Cheng J (2018). Targeting pericytes for therapeutic approaches to neurological disorders. Acta Neuropathol..

[90] Hesp ZC (2018). Proliferating NG2-cell-dependent angiogenesis and scar formation alter axon growth and functional recovery after spinal cord injury in mice. J. Neurosci..

[91] Starting the scar: a primary role for pericytes? *Nat. Med.* **17**, 1052–1053 (2011). 10.1038/nm0911-1052 21900921

[92] Dias DO (2018). Reducing pericyte-derived scarring promotes recovery after spinal cord injury. Cell.

[93] Caporali A (2017). Contribution of pericyte paracrine regulation of the endothelium to angiogenesis. Pharm. Ther..

[94] Yamadera M (2015). Microvascular disturbance with decreased pericyte coverage is prominent in the ventral horn of patients with amyotrophic lateral sclerosis. Amyotroph. Lateral Scler. Frontotemporal Degener..

[95] Rozycka J, Brzoska E, Skirecki T (2017). Aspects of pericytes and their potential therapeutic use. Postepy Hig. Med Dosw (Online).

[96] Katare R (2011). Transplantation of human pericyte progenitor cells improves the repair of infarcted heart through activation of an angiogenic program involving micro-RNA-132. Circ. Res..

[97] Tachibana M, Yamazaki Y, Liu CC, Bu G, Kanekiyo T (2018). Pericyte implantation in the brain enhances cerebral blood flow and reduces amyloid-beta pathology in amyloid model mice. Exp. Neurol..

[98] Coatti GC (2017). Pericytes extend survival of ALS SOD1 mice and induce the expression of antioxidant enzymes in the murine model and in IPSCs derived neuronal cells from an ALS patient. Stem Cell Rev..

[99] Winkler EA, Bell RD, Zlokovic BV (2011). Central nervous system pericytes in health and disease. Nat. Neurosci..

[100] Oh JS (2010). Hypoxia-preconditioned adipose tissue-derived mesenchymal stem cell increase the survival and gene expression of engineered neural stem cells in a spinal cord injury model. Neurosci. Lett..

[101] Ding WG, Yan WH, Wei ZX, Liu JB (2012). Difference in intraosseous blood vessel volume and number in osteoporotic model mice induced by spinal cord injury and sciatic nerve resection. J. Bone Min. Metab..

[102] Yokota K (2017). Periostin promotes scar formation through the interaction between pericytes and infiltrating monocytes/macrophages after spinal cord injury. Am. J. Pathol..

[103] Hill J, Rom S, Ramirez SH, Persidsky Y (2014). Emerging roles of pericytes in the regulation of the neurovascular unit in health and disease. J. Neuroimmune Pharm..

[104] Tran AP, Warren PM, Silver J (2018). The biology of regeneration failure and success after spinal cord injury. Physiol. Rev..

[105] Ehm O (2010). RBPJkappa-dependent signaling is essential for long-term maintenance of neural stem cells in the adult hippocampus. J. Neurosci..

[106] Shibata T (1997). Glutamate transporter GLAST is expressed in the radial glia-astrocyte lineage of developing mouse spinal cord. J. Neurosci..

[107] Wynn TA (2007). Common and unique mechanisms regulate fibrosis in various fibroproliferative diseases. J. Clin. Investig..

[108] Narang A, Zheng B (2018). To scar or not to scar. Trends Mol. Med..

[109] Dias DO, Goritz C (2018). Fibrotic scarring following lesions to the central nervous system. Matrix Biol..

[110] Chim SM (2013). Angiogenic factors in bone local environment. Cytokine Growth Factor Rev..

[111] Zhu S (2020). Endothelial cells produce angiocrine factors to regulate bone and cartilage via versatile mechanisms. Theranostics.

[112] Lamagna C, Bergers G (2006). The bone marrow constitutes a reservoir of pericyte progenitors. J. Leukoc. Biol..

[113] Tang W (2008). White fat progenitor cells reside in the adipose vasculature. Science.

[114] Krautler NJ (2012). Follicular dendritic cells emerge from ubiquitous perivascular precursors. Cell.

[115] Zhao H (2014). Secretion of shh by a neurovascular bundle niche supports mesenchymal stem cell homeostasis in the adult mouse incisor. Cell Stem Cell.

[116] Diaz-Flores, L., Gutierrez, R., Lopez-Alonso, A., Gonzalez, R. & Varela, H. Pericytes as a supplementary source of osteoblasts in periosteal osteogenesis. *Clin. Orthop. Relat. Res.* 280–286 (1992). 1735226

[117] Doherty MJ (1998). Vascular pericytes express osteogenic potential in vitro and in vivo. J. Bone Min. Res..

[118] Supakul, S. et al. Pericytes as a source of osteogenic cells in bone fracture healing. *Int. J. Mol. Sci.* **20**, 1079 (2019). 10.3390/ijms20051079 PMC6429337 30832329

[119] Meyers CA (2018). Early immunomodulatory effects of implanted human perivascular stromal cells during bone formation. Tissue Eng. Part A.

[120] Lee S (2015). Brief report: human perivascular stem cells and nel-like protein-1 synergistically enhance spinal fusion in osteoporotic rats. Stem Cells.

[121] James AW (2012). An abundant perivascular source of stem cells for bone tissue engineering. Stem Cells Transl. Med..

[122] James, A. W. et al. Use of human perivascular stem cells for bone regeneration. *J. Vis. Exp.* e2952 (2012). 10.3791/2952 PMC3466949 22664543

[123] Askarinam A (2013). Human perivascular stem cells show enhanced osteogenesis and vasculogenesis with Nel-like molecule I protein. Tissue Eng. Part A.

[124] Chung CG (2014). Human perivascular stem cell-based bone graft substitute induces rat spinal fusion. Stem Cells Transl. Med..

[125] Tawonsawatruk T (2016). Adipose derived pericytes rescue fractures from a failure of healing–non-union. Sci. Rep..

[126] James AW (2017). Pericytes for the treatment of orthopedic conditions. Pharm. Ther..

[127] James AW, Peault B (2019). Perivascular mesenchymal progenitors for bone regeneration. J. Orthop. Res..

[128] Baker AH, Peault B (2016). A Gli(1)ttering role for perivascular stem cells in blood vessel remodeling. Cell Stem Cell.

[129] Jotereau FV, Le Douarin NM (1978). The development relationship between osteocytes and osteoclasts: a study using the quail-chick nuclear marker in endochondral ossification. Dev. Biol..

[130] Salazar VS, Gamer LW, Rosen V (2016). BMP signalling in skeletal development, disease and repair. Nat. Rev. Endocrinol..

[131] Ahi EP (2016). Signalling pathways in trophic skeletal development and morphogenesis: Insights from studies on teleost fish. Dev. Biol..

[132] Diaz-Flores L (2012). Cell sources for cartilage repair; contribution of the mesenchymal perivascular niche. Front. Biosci..

[133] Grcevic D (2012). In vivo fate mapping identifies mesenchymal progenitor cells. Stem Cells.

[134] Diaz-Flores L, Gutierrez R, Gonzalez P, Varela H (1991). Inducible perivascular cells contribute to the neochondrogenesis in grafted perichondrium. Anat. Rec..

[135] Zhang X (2011). The Nell-1 growth factor stimulates bone formation by purified human perivascular cells. Tissue Eng. Part A.

[136] Rajashekhar G (2008). IFATS collection: adipose stromal cell differentiation is reduced by endothelial cell contact and paracrine communication: role of canonical Wnt signaling. Stem Cells.

[137] Meury T, Verrier S, Alini M (2006). Human endothelial cells inhibit BMSC differentiation into mature osteoblasts in vitro by interfering with osterix expression. J. Cell Biochem..

[138] Mankin HJ (1992). Nontraumatic necrosis of bone (osteonecrosis). N. Engl. J. Med..

[139] Mafi R, Hindocha S, Mafi P, Griffin M, Khan WS (2011). Sources of adult mesenchymal stem cells applicable for musculoskeletal applications - a systematic review of the literature. Open Orthop. J..

[140] Papakostidis C, Tosounidis TH, Jones E, Giannoudis PV (2016). The role of “cell therapy” in osteonecrosis of the femoral head. A systematic review of the literature and meta-analysis of 7 studies. Acta Orthop..

[141] Slobogean GP, Sprague SA, Scott T, Bhandari M (2015). Complications following young femoral neck fractures. Injury.

[142] Gangji V, Toungouz M, Hauzeur JP (2005). Stem cell therapy for osteonecrosis of the femoral head. Expert Opin. Biol. Ther..

[143] Zhao L, Kaye AD, Kaye AJ, Abd-Elsayed A (2018). Stem cell therapy for osteonecrosis of the femoral head: current trends and comprehensive review. Curr. Pain. Headache Rep..

[144] Li R (2018). Stem cell therapy for treating osteonecrosis of the femoral head: From clinical applications to related basic research. Stem Cell Res. Ther..

[145] Mao L (2020). Efficacy and safety of stem cell therapy for the early-stage osteonecrosis of femoral head: a systematic review and meta-analysis of randomized controlled trials. Stem Cell Res. Ther..

[146] Cordova LA (2016). Severe compromise of preosteoblasts in a surgical mouse model of bisphosphonate-associated osteonecrosis of the jaw. J. Craniomaxillofac Surg..

[147] Deyo RA, Gray DT, Kreuter W, Mirza S, Martin BI (2005). United States trends in lumbar fusion surgery for degenerative conditions. Spine.

[148] Covas DT (2008). Multipotent mesenchymal stromal cells obtained from diverse human tissues share functional properties and gene-expression profile with CD146^+^ perivascular cells and fibroblasts. Exp. Hematol..

[149] Russell KC (2013). Cell-surface expression of neuron-glial antigen 2 (NG2) and melanoma cell adhesion molecule (CD146) in heterogeneous cultures of marrow-derived mesenchymal stem cells. Tissue Eng. Part A.

[150] Fattal E, Kassinos SC (2018). Editorial on the special issue “SimInhale”. Eur. J. Pharm. Sci..

[151] Iacobaeus E (2017). Dynamic changes in brain mesenchymal perivascular cells associate with multiple sclerosis disease duration, active inflammation, and demyelination. Stem Cells Transl. Med..

